# Stress and Coping During an HIV Cure-Related Trial with an Analytical Treatment Interruption: A Qualitative Assessment of the Experiences of Young Women in Durban, South Africa

**DOI:** 10.1177/23259582261423985

**Published:** 2026-02-13

**Authors:** Ali Ahmed, Miranda Hill, Krista L. Dong, Mzwakhe Wiseman Ngcobo, Ayanda Zulu, Ntombifuthi Langa, Luyanda Maphalala, Vanessa Pillay, Maud Mthembu, Whitney Tran, Rachel Lau, Jamila K. Stockman, Thumbi Ndung’u, Karine Dubé

**Affiliations:** 1 Division of Infectious Diseases and Global Public Health, School of Medicine, 21814 University of California, San Diego (UCSD), La Jolla, CA, USA; 2 Department of Obstetrics, Gynecology, and Reproductive Sciences, School of Medicine, University of California, San Francisco (UCSF), San Francisco, CA, USA; 3 Ragon Institute of Mass General Brigham, MIT, and Harvard, Cambridge, MA, USA; 4 Infectious Diseases Division, Massachusetts General Hospital, Boston, MA, USA; 5 Harvard Medical School, Cambridge, MA, USA; 6 Integration of TB in Education and Care for HIV/AIDS (ITEACH), Pietermaritzburg, South Africa; 7 Females Rising through Education, Support and Health (FRESH), Durban, South Africa; 8 School of Applied Human Sciences, Department of Social Work, University of KwaZulu-Natal (UKZN), Durban, South Africa; 9 HIV Pathogenesis Programme (HPP), The Doris Duke Medical Research Institute, UKZN, Durban, South Africa; 10 Africa Health Research Institute (AHRI), Durban, South Africa; 11 Division of Infection and Immunity, University College London, London, UK

**Keywords:** women living with HIV, HIV cure research, resource-limited settings, PrEP, mental health, HIV prevention

## Abstract

**Introduction:**

Young women in sub-Saharan Africa bear a disproportionate HIV burden yet rarely participate in cure-related studies. Analytical treatment interruptions (ATI), used to assess sustained control off therapy, raise clinical, ethical, and psychosocial concerns.

**Methods:**

We conducted a longitudinal qualitative study within a Phase 2A ATI trial (NCT05281510) at the FRESH site in Durban, South Africa. Nineteen women living with HIV (median age 26) completed in-depth interviews at 4 timepoints. We applied framework analysis informed by the Lazarus-Folkman stress and coping model.

**Results:**

Participants enrolled to contribute to science, reduce pill burden, and due to trust in the clinical team. They anticipated viral rebound, resistance, stigma, and partner transmission. Over time, many reported improved emotional well-being, using meaning-based strategies (pride in contribution) and problem-focused strategies (self-monitoring, condom negotiation). Burdens included stigma, selective disclosure, partner resistance, frequent visits, and blood draws. Benefits included increased HIV literacy, self-management, and comfort with procedures.

**Conclusion:**

ATI-inclusive clinical trials can be acceptable when designs include clear and ongoing education, strong confidentiality protections, mental health and peer support, partner-inclusive risk reductions, and flexible scheduling with practical supports to minimize participation burden and potential psychosocial harms.

**Clinical trial registration:**

NCT05281510

## Plain Language Summary

Stress and Coping in Young Women During an HIV Cure-Related Trial in Durban, South Africa

Young women in sub-Saharan Africa are disproportionately affected by HIV, yet they are underrepresented in HIV cure-related studies that include a temporary pause in treatment. This study explored the experiences of 19 young women in Durban, South Africa who participated in a Phase 2A HIV cure-related trial that included an analytical treatment interruption (ATI) under close clinical monitoring. Across interviews conducted from screening through end of the study, participants described specific stressors and shifting views of risk. The ATI period was marked by uncertainty, worry about viral rebound and health impacts, concerns about stigma and unintended disclosure, and fear of transmitting HIV to partners. Participants also described practical burdens from frequent clinic visits and monitoring. Over time, many reported increased confidence as they better understood study procedures and their health indicators, along with improved HIV knowledge and self-management. Participants described coping strategies supported by trusted clinical relationships, social support, planning around visits, and negotiating safer practices. Overall, participants indicated that ATI studies can be acceptable when they include clear and repeated education, strong confidentiality protections, access to mental health and peer support, and flexible scheduling and practical supports that reduce participation burden.

## Introduction

In KwaZulu-Natal (KZN), South Africa, young women face an extraordinarily high risk of HIV acquisition. For example, in 1 community, over 60% of women between 25 and 44 years old are living with the virus.^1,2^ While antiretroviral therapy (ART) offers sustained viral suppression, it is not curative, driving global efforts to develop interventions aimed at sustained ART-free control or cure.^3^ Many HIV cure-related trials rely on an analytical treatment interruption (ATI), a closely monitored pause of ART, to assess whether an intervention can stimulate durable viral control in the absence of therapy.^4–7^ ATIs have become a cornerstone of HIV cure research in the absence of a validated biomarker that can predict viral rebound following cessation of therapy.^8,9^ To date, most ATI-inclusive trials have been conducted in high-income countries enrolling primarily men, excluding women and cure research in regions with the greatest HIV burden where interventions stand to have the greatest impact.^10,11^ In 2025, the first ATI-inclusive HIV cure-related trial conducted in Africa was completed, and enrolled exclusively women recruited from the Females Rising through Education, Support, and Health (FRESH) clinical research site in Durban, South Africa.^1,10,12–14^

ATIs offer essential scientific insight but introduce ethical and psychosocial complexities.^15,16^ By providing informed consent to temporarily pause their treatment, participants accept health risks associated with viral rebound, including the possibility of transient high-level viremia and immunological decline before ART is restarted.^17,18^ Specific criteria for ART resumption (eg, if viral load or CD4+ counts reach predefined thresholds) and frequent clinical monitoring are relied upon to protect participants' safety.^19,20^ Nevertheless, deliberate HIV treatment discontinuation can provoke anxiety and ambivalence among participants.^10,17,21,22^ Participants must balance the incentive of contributing toward finding an HIV cure against their fears of personal health risks.^10^ Studies have found substantial psychosocial strain among people considering ATI trials, with high rates of anxiety and depressive symptoms before stopping ART.^23–25^

Beyond risks to themselves, participants in ATI trials must also consider the risk of transmission to their partners.^26^ Participants are encouraged to choose from multiple strategies to mitigate HIV transmission risks during the ATI, including disclosure, abstinence, condom use, and encouraging partners without HIV to use pre-exposure prophylaxis (PrEP).^27^ ATIs may require women to make difficult decisions that involve emotional strain, social pressure, and risk of gender-based violence, contributing toward uncertainty about trial participation.^17^ For many women, especially in settings with unequal gender dynamics and limited access to psychosocial support services, applying these measures can be overwhelming.^28^ Some avoid suggesting condom use and disclosure of their HIV status due to fear of rejection, loss of support, or judgment.^29^ In several cases, women do not disclose their HIV status prior to trial enrollment, posing an ethical conundrum around pausing treatment.^30–32^ Conversely, trust in the research team and frequent monitoring are often cited as factors that help participants feel more confident about undertaking an ATI.^23^ These nuanced dynamics highlight the need for studies to better understand how ATIs contribute to participants' stress, and support services and interventions that should be incorporated into ATI trial design to lessen potential detrimental outcomes.^24^

To ensure that HIV cure trials are safe and relevant to the populations most affected by HIV, it is crucial to understand how participants experience and navigate stress throughout the process of stopping and restarting ART.^18^ Despite the disproportionate burden of HIV among young women in high-prevalence settings and the unique risks they might experience during ATI trials, women have been largely excluded from HIV cure trials. There is limited empirical evidence on the psychosocial experiences of women living with HIV (WLHIV), particularly by those living in settings with limited access to psychosocial support services, gender inequality, and high rates of gender-based violence.^33,34^ To address this gap, we conducted a longitudinal qualitative study nested within a Phase 2A ATI trial (ClinicalTrials.gov NCT05281510) in Durban, South Africa, that enrolled WLHIV. Guided by the transactional model of stress and coping, we examined how participants appraised psychosocial and biomedical stressors, risks, and benefits across 4 trial timepoints, with particular attention to ATI and ART resumption. We identified the coping strategies and supports participants used and recommended to manage stress and mitigate potential psychosocial harms.^35^ We aimed for these findings to inform participant-centered designs of future ATI inclusive HIV cure-related trials that enroll women, particularly in sub-Saharan Africa and similar HIV high-burden, resource-constrained settings.

## Methods

### Study Design and Setting

**Figure 1.**
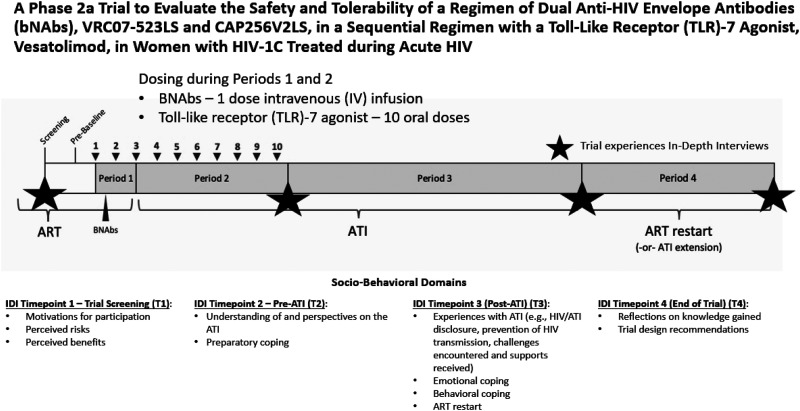
Longitudinal in-depth interviews nested within the phase 2A ATI trial. The schematic shows 4 trial periods and key milestones, including ART, intervention dosing during Periods 1 and 2, analytical treatment interruption (Period 3), and ART restart or ATI extension (Period 4). Dosing points are labeled on the timeline as numbered inverted triangles (1 to 10) for the 10 oral vesatolimod doses and a solid triangle for the single intravenous infusion of dual bNAbs (VRC07-523LS and CAP256V2LS). Stars indicate qualitative interview timepoints at screening (T1), pre-ATI (T2), post-ATI (T3), and end of trial (T4). Abbreviations: ATI, analytical treatment interruption; ART, antiretroviral therapy; bNAbs, broadly neutralizing antibodies.

Between June 2022 and January 2025, we conducted a longitudinal, qualitative socio-behavioral research (SBR) study nested within an ATI-inclusive HIV cure-related clinical trial. The study used qualitative methods to examine participants’ perceived trial-related risks and psychosocial impacts across 4 key timepoints during the trial. Longitudinal interviews enabled capture of the depth and complexity of their emotions, decision-making, and contextual influences essential to understanding their lived experiences during the trial.^36^ The SBR study ran concurrently with the clinical trial, a Phase 2A HIV cure-related trial (NCT05281510) conducted at the FRESH clinical research site in Durban, South Africa.^1,37^ The SBR study required volunteers to complete 4 in-depth interviews (IDIs) at key time points during the trial: T1 (trial screening or baseline), T2 (immediately before ATI), T3 (at ART resumption or continued viral control), and T4 (end of trial) (Figure 1). The FRESH program is a well-established research platform that combines basic science research with empowerment initiatives that include HIV prevention, provision of PrEP, life and job-skills training.^14^ FRESH enrolls young women at higher risk of HIV acquisition who have limited opportunity for economic independence into a 9-month program that includes intensive HIV prevention counseling, access to free PrEP, and twice-weekly viral load testing. Women who acquire HIV are given access to immediate ART and long-term follow-up that includes holistic, women-centered psychosocial support.^14,38^

### Participants and Recruitment

Young women enrolled in the ATI-inclusive trial were recruited to join the SBR study. We applied the parent clinical trial eligibility criteria (ClinicalTrials.gov NCT05281510). We enrolled adult women from the FRESH cohort who had maintained virologic suppression on ART for at least 12 months, met protocol laboratory thresholds, and had documented viral sensitivity to VRC07-523LS or CAP256V2LS. We excluded participants who were pregnant or breastfeeding, had HBV or HCV infection, required immunosuppression or had recent immunomodulatory exposures, had prior HIV vaccine or monoclonal antibody exposure, had an acute illness, had substance use that could compromise safety or adherence, or had a serious medical or psychiatric condition that could interfere with participation. Trial visits included product dosing with an extended post-dose observation period. Optional invasive procedures included lymph node excision and distal colon biopsies. We obtained separate written informed consent for the SBR study, confirming willingness to complete the 4 IDIs.

The FRESH clinical research team introduced participants to the qualitative study at the trial screening visit. Interested participants were referred to the independent SBR study team who obtained written informed consent separate from the clinical trial. Participation in the qualitative study did not affect eligibility for or continuation in the trial. To protect confidentiality, all SBR participants were assigned an anonymized, study-specific code. Participants received 150 South African rands (approximately $9 USD) per interview to compensate for time and travel. In recognition of potential psychosocial impact of participating in the interviews, including triggering prior trauma, participants were provided access to African-centered psychosocial support if needed.

### Interview Guide

We developed and refined the in-depth interview guide through iterative team discussions and expert input to ensure cultural and contextual appropriateness for young WLHIV. The transactional theory of stress and coping informed the development of the interview guide. The guide was designed to explore key dimensions of participants’ experiences with stress and coping during the trial, including motivations for trial participation; perceived risks and benefits, understanding of and perspectives on ATIs, HIV and ATI-related disclosure, HIV transmission prevention, and additional challenges and sources of support. Questions were open-ended and included probes to elicit depth and consistency across interviews. The guide was tailored for each of the 4 interview timepoints to capture changes in experiences over the course of the trial. The interview guide can be made available upon request to the corresponding author.

### Data Collection

Following written informed consent, participants completed a demographic questionnaire. IDIs were administered by trained SBR study staff in a private room at the FRESH clinical research site. Interviews were conducted in participants’ preferred language (eg, English, *isi*Zulu or a mix of both), audio-recorded, transcribed verbatim, and translated into English. Interviews lasted 30-90 minutes. Our transcription approach employed naturalized transcription in order to honor the voices of the participants. Finally, although we did not conduct participant member checking, we implemented alternative validation strategies (eg, double-checking transcripts and team-based review) to support analytic rigor.

### Data Analysis

Upon interview completion, audio recordings were stored on a secure, team-only drive, and verified for quality by research team members (M.W.N., A.Z., and N.L.). To analyze the qualitative data, we used framework analysis in 4 iterative stages: familiarization, indexing, charting, and synthesis to blend inductive insights with deductive structure.^39^ First, 3 coders (A.A., W.T., and R.L.) immersed themselves in the transcripts to capture initial impressions. We began with an *a priori* codebook based on the transactional model of stress and coping, focusing on primary and secondary appraisals as well as problem-focused and emotion-focused responses.^35^ Through regular team debriefings, the codebook was refined to incorporate trial-specific themes that emerged from the data, such as anxiety about condom negotiation and discomfort with trial procedures. We did not use data saturation to determine sample size because the SBR study was nested within a parent clinical trial with a predefined cohort. We assessed thematic sufficiency during analysis by tracking the emergence of new codes and confirmed codebook stability across participants and timepoints. During indexing, coders independently applied and reconciled codes until reaching consensus. We did not calculate formal intercoder agreement metrics, as our emphasis was on collaborative interpretation and consensus-building rather than statistical concordance, consistent with reflexive and interpretive qualitative methods.^40^ We then charted coded data into a framework matrix organized by participant and timepoint, which allowed us to examine within-participant trajectories across T1 to T4 and to compare patterns across participants. In synthesis, we identified higher-order themes that explained how stress appraisals shifted over time, what resources participants drew upon, and how coping strategies adapted across trial phases. We also examined divergent accounts and contrasting cases to refine interpretations and avoid overgeneralization. Following coding, social and behavioral scientists (K.Du. and M.H.) reviewed the resulting codes and contributed additional insights to the thematic development. To explore whether coping trajectories differed by time to ART resumption, we compared study findings between the trial's 3 ATI outcomes groups: early ART restart (ER; <16 weeks, *n* = 6), delayed ART restart (DR; 16-44 weeks, *n* = 7), and long-term delayed ART restart (LTDR; >44 weeks, *n* = 6) groups. We conducted thematic comparisons across these subgroups to examine potential variations in stress appraisal and coping strategies.

We report this qualitative study in accordance with the Consolidated Criteria for Reporting Qualitative Research (COREQ) checklist,^41^ which we include as a Supplementary File 1.

### Ethics Statement

The University of KwaZulu-Natal Biomedical Research Ethics Committee (00002897/2021) was the Institutional Review Board (IRB) of record. In addition, the IRBs of Mass General Brigham (protocol #2022P000729) and the University of California San Diego (UCSD) (#806466) reviewed the study.

## Results

We enrolled 19 WLHIV (ages 23-32 years; median 23, IQR 5.3) in this SBR study; one declined participation. All participants spoke isiZulu, lived in Durban, and had male sexual partners. Eight completed secondary education (median highest grade 10), and 17 were unemployed. Seventeen lived with family (median household size 5); 13 had children, and 3 cared for a child under 2 years. Aside from the FRESH trial, none had participated in a prior clinical trial (Table 1).

**Table 1.** Participants’ Sociodemographic Characteristics (Durban, South Africa, 2022-2025).

Characteristics	*n* (%)	Median	IQR
*Sociodemographics*			
Age		23.0	5.3
Gender of intimate partners			
Male	20 (100.0)		
Home language^a^			
Zulu	20 (100.0)		
Primary residence			
Umlazi	20 (100.0)		
Religious affiliation			
None	1 (5.0)		
Christian	15 (75.0)		
Traditionalist	3 (15.0)		
No response	1 (5.0)		
Education			
No school	1 (5.0)		
Finished grade but not matriculated	5 (25.0)		
Matriculated	8 (40.0)		
Tertiary	6 (30.0)		
If finished grade but not matriculated, which grade did you finish?		10	1.0
Employment			
Unemployed	17 (85.0)		
Employed part-time informally	1 (5.0)		
Self-employed/own business	2 (10.0)		
Who lives with you^b^			
With roommate(s)	1 (0.0)		
With partner (boyfriend/girlfriend, husband/wife)	4 (0.0)		
With family	17 (90.0)		
Number of people in household		5	3.0
Number of children		1	1.0
Sole/primary caregiver			
Child below 2 years of age	3 (15.0)		

Abbreviations: *N*, sample size; IQR, interquartile range.

a The total number of responses do not equal *n* = 20, as participants could pick more than one home language.

b The total number of responses do not equal *n* = 20, as participants could pick more than one response for who lives with you.

### Psychosocial Stressors (T1-T4)

At the trial start, many participants expressed heightened emotions, including a sense of vulnerability due to psychosocial challenges, fear of becoming sick due to viral rebound, and transmitting HIV to their sex partner.

While we are in this process of pausing my ARV's [antiretrovirals] because my virus is still suppressed … what if it wakes up and starts to spread and then I become sick? This is what I am afraid of and if I end up infecting [passing HIV to] my partner? (T1-G050)

Others worried about the emotional strain of managing conversations about their trial participation, especially due to frequent trial visits and following trial procedures that could draw attention and risk unintended HIV disclosure. Participants also anticipated psychosocial risks, particularly the emotional strain of explaining their trial involvement and fatigue of repeatedly negotiating prevention measures with partners.

The issue of … having to tell people that I am busy with this and that … after doing lymph nodes, you do not feel good so people might ask what is happening, now I will have to explain myself. (T1-G061)

Despite these worries, several participants described trusting the research staff and feeling well-informed, which helped mitigate perceived risks but did not mitigate fears about viral rebound.

Well, there's not much risk … they've told me everything, and I understood everything that they told me. So, the only risk that I can say one can face when they're involved in the study, is the one that involves the ATI period where the virus could spiral out of control. (T1-G040)

Participants described a range of challenges related to the trial, which fell into 3 main categories: (1) interpersonal and disclosure-related challenges, (2) trial-related procedures, and (3) logistical and scheduling issues. These challenges were often mitigated by supportive clinical research staff, flexible accommodation, and adaptive coping strategies.

#### Interpersonal and Disclosure-Related Challenges

Participants navigated sensitive conversations with family and partners related to HIV status, trial participation, and the ATI. Disclosure was often selective, encouraged by onsite counsellors who recommended disclosure to at least 1 trusted person. For some participants, disclosure brought about conflict or being judged; 1 HIV disclosure to a partner led to the relationship ending. Others feared their partner would blame them if they learned of their HIV status. One participant experienced stress after choosing to abstain from alcohol while participating in the trial.

Personally, the challenge I faced was having to tell my family about the study … [instead] I chose just one person that I trust and explained everything to them including that I am not comfortable to tell the rest of the family … that really helped … I was able to talk to my older sister and explained everything to her. (T4-G040)

The challenge I had was telling my partner my status … we ended up breaking the relationship. (T4-G021)

#### Challenges Related to Trial Procedures

Some participants experienced headaches or anxiety related to trial procedures. Venipuncture was a frequent concern, when repeated blood draws led to soreness or bruising. A few worried about the volume of blood sampled as well as the possible cumulative effect of frequent sampling, though most viewed these issues as temporary and noted feeling that they were managed effectively by research staff.

Another thing, it is the time they withdraw blood, they took a lot of it. My arm was painful, it became green for 2 weeks … I did tell the nurses, but because they had already started taking the blood, it was not easy to stop. (T2-G051)

Less frequent optional procedures, such as lymph node excision and gut biopsies, were also described as stressful. In a rare event, 1 participant recounted a prolonged recovery after an optional biopsy site became infected, requiring additional treatment and functional limitations. While such events are uncommon, they can occur and underscore the importance of clear risk information, close post-procedure monitoring, and responsive care.

I stayed (in the hospital) for seven days and had a [procedure] followed by wound therapy for a hole in my leg. I had to carry the wound therapy device with me everywhere I went, and I had to visit [a physician] twice a week. (T3-G010)

#### Logistical and Scheduling Challenges

Some participants encountered practical barriers to trial visit attendance, such as overlapping responsibilities or academic commitments. In these cases, the clinical research team's flexibility helped maintain trial participation. For example, when a trial visit conflicted with an online exam for school, the research site allowed the participant access to a laptop to complete her exam during the trial visit.

I had an exam to write, and the same day was my visit for FRESH. I told them and they handled it very well … they gave me a laptop to write. (T2-G051)

### Evolving Appraisals of Stressors, Risks, and Benefits (T1-T4)

Participants revealed an evolving emotional and cognitive trajectory shaped by their engagement in the ATI trial.

#### Motivational Relevance at Screening (T1)

A multidimensional motivational profile emerged at trial screening. Trial participants most frequently invoked collective altruism, describing trial participation as a pathway to accelerate HIV cure science for the benefit of peers, especially other young women and future generations.

I basically agreed to do it because I felt like I’d be contributing to the greater good when it comes to bettering people’s lives and finding the HIV vaccine or cure like they [are] expecting. (T1-G040)

Many participants described the burdens of daily ART fatigue, forgetfulness, and HIV-related stigma as reasons for hoping the trial might lead to an alternative option to reduce the treatment burden. For these participants, the appeal of pausing or replacing daily medication was both practical and symbolic.

Taking treatment every day is a headache and the stress of forgetting … so if there is a possibility … I found myself willing to be in that place where I will not have to take medication at all. (T1-G060)

#### The Pivotal Role of Social Support

Some participants described their decision to join the trial as a rare opportunity to gain knowledge and emotional support, talk about their HIV status, receive counseling, or build confidence to enable them to disclose their HIV status to family or partners. Although financial incentives were frequently mentioned, they were typically described as helpful additions rather than primary motivators. Nearly all participants described the decision to participate as one they made independently, often rooted in a trusting relationship with the clinical trial team at FRESH. A few participants consulted family members or partners about their decision to participate, but most emphasized their own agency and understanding at the time of trial screening.

By the ATI start on trial day 35 (T2), participants began to rely on available support and behavioral modification to manage risk, including seeking onsite counseling, symptom reporting to the clinical research team, and engaging in safer sex practices. For some, trial participation provided a sense of purpose, with access to HIV-related knowledge and support services that were otherwise unavailable.

The study kept me busy since I wasn’t doing anything at the time. The best thing is I am getting [a] stipend, learning about the virus, like my viral load, and if ever there is a problem I should phone … or I just take a taxi to FRESH to come and explain. (T2-G030)

Supportive relationships helped alleviate fears for some trial participants. A few described family members or partners who not only encouraged their trial participation but also provided active support, including visiting the FRESH site, asking about the trial, and speaking to trial staff about procedures. This active engagement reinforced trust and reduced the emotional burden of participation.

I told my partner [about the trial], and he was shocked at first. But then we went through the consent form together. He asked questions, and eventually he said if I am comfortable and I trust the FRESH team, then he supports me. That made me feel better. (T1-G071)

My boyfriend actually came with me one day to the clinic. He wanted to ask the nurses himself. After that, he was more at peace with everything. (T2-G020)

#### Feeling Valued, Giving Forward

When participants met ART re-initiation criteria (T3), they reported feeling more informed and self-assured. Many developed a deeper understanding of their health, appreciated the individualized, attentive care at the FRESH site, and began to see themselves as part of a collective scientific effort.

I got huge knowledge … I’ve learnt many things … because here is not the same as public clinics, where they just draw blood, give you pills, and you go. They do not practice this kind of care that they have here at FRESH. I was so happy, waking up and knowing that [I was] going to get information. (T3-G090)

By the final trial visit (T4), participants described personal growth, resilience, and transformation in self-identity from living with HIV to becoming an informed contributor to HIV cure research. The responsive clinical monitoring provided throughout the study, including pregnancy prevention support and PrEP for partners, reinforced trust in the research team and a strong sense of being valued as a trial participant.

What we are doing is no longer just about us but for those who will come after us … in the hope that a cure will be found. I also benefited from the [trial] because they ensured that we took measures to prevent pregnancy and provided us with prevention methods like PrEP and support. (T4b-G091)

### Stress Appraisals, ATI, and Art Restart (T2-T4)

#### ATI: Heightened Anxiety and Risk Appraisal

Participants described a nuanced emotional and cognitive journey throughout the ATI and ART restart periods, shaped by initial anxieties and subsequent adaptations. At T2, many participants expressed concentrated fear about stopping ART, despite reassurances against long-standing messaging around lifelong HIV treatment adherence. The fear of interrupting HIV treatment included biomedical risks related to viral rebound and development of ART resistance. There were additional fears about potential side effects from the trial's investigational products, emotional vulnerability from not knowing the trial outcomes, psychosocial stress associated with not disclosing trial participation, and fear of HIV-related stigma associated with disclosure. Participants often framed ATI as a moment when control shifted away from their usual routines and into a more uncertain space, where they had to weigh trust in the clinical trial against deeply learned messages that daily ART kept them safe and responsible. In this context, stress appraisal was not limited to clinical risk; it also reflected moral and relational concerns about being a “good” partner and protecting one's future.

I took pills every day. Now if I was stopping … I was worried about the side effects of these study drugs and if my body will respond well or not, I am very scared, thinking about the virus growing inside me again … I felt like I was gambling with my health. (T2-G060)

Several participants used language of chance, loss, or danger, suggesting that fear was not abstract, but closely tied to anticipated consequences for their health, identity, and future plans.

We never know when the viral load will increase, so we must minimize sex and alcohol. But my main concern is spiritual and emotional health, if that's not okay, it can affect you physically, even if you follow all the doctor's orders. That's what I experienced last time. (T2-G090)

Participants also emphasized holistic appraisals of risk. Some linked emotional well-being, stress, and spiritual stability to physical outcomes, and treated ATI as a period requiring heightened self-regulation across multiple domains. This framing positioned coping as both behavioral, such as avoiding alcohol and reducing sex, and emotional, such as protecting one's “heart” and maintaining calm to prevent distress from escalating into physical symptoms or perceived vulnerability.

#### Nondisclosure: Temporary Relief, New Challenges

For participants who had not disclosed their HIV status, the ATI offered temporary relief from the emotional burden of hiding their HIV medications. However, ATIs also introduced new challenges, as they imply disclosure of HIV status to partners.

In my view it is alright because I am not living alone, and it becomes a challenge when it is time to take my medication. I always hide them because I cannot disclose them, maybe they will gossip about me. So, I think the ATI phase will be good for me, nothing will worry me. (T2-G051)

This relief reflected more than convenience, it functioned as an emotional respite from constant vigilance and fear of stigma. At the same time, several participants noted that ATI did not remove disclosure stress; it shifted it. They worried that increased clinic visits, behavior changes, or later ART restart could trigger questions, requiring new strategies to protect privacy.

#### Uncertain Timelines: From Helplessness to Measured Trust

Participants expressed being anxious and helpless due to unclear timelines for ART restart and fluctuating viral load results, which evoked fears of uncontrolled HIV and past experiences of HIV treatment failure in the community. Gradually, consistent clinical monitoring and seeing their viral load remain suppressed without ART allowed some to move from fear toward a more measured sense of trust and control.

I was very stressed. One day they said my viral load was going down and I felt hopeful, then suddenly it increased again. That's when I started worrying what might happen to my body, especially after seeing what happened to others who defaulted. (T2-G080)

This quote illustrates how past observations shaped present appraisal. Participants did not interpret viral load changes in isolation; they interpreted them through lived and witnessed experiences, which intensified anxiety and reduced perceived control when results fluctuated.

Even though they explained everything and said there's no guarantee* … *like medication [experimental product], hasn't been approved yet, I was really scared. But as I kept coming in every week, and they were showing me the differences in my viral load and showing me that it was still being suppressed even though I wasn’t taking my ARVs [antiretroviral treatment] anymore, I started to relax. (T2-G060)

Regular monitoring operated as a key resource that strengthened secondary appraisal, increasing perceived safety and control even when uncertainty remained. Over time, some participants described learning to tolerate ambiguity, using repeated feedback from clinical visits as evidence that risk was being managed, which enabled steadier coping.

#### ART Restart (T3): Relief, Grief, and Acceptance

Trial participants expressed a range of emotional responses to restarting ART (T3). Some noted feeling relief and reassurance, viewing ART as protective and a return to normalcy, while others experienced disappointment or grief, particularly those who had hoped for a longer period off ART. Many understood the limitations of the trial, accepting the need to resume ART as part of the process, even when it was emotionally difficult.

When they said I should start [ART] again, I was relieved. I felt like I had my weapon back. (T3-G020)

Participants who described ART as a “weapon” framed restart as protection and regained agency, which helped them recommit to pill taking and return to established self-care practices.

I had mixed emotions … happy but also with doubts and hope … Restarting ARVs wasn’t a problem … I protect my heart so I’m not too disappointed if things go wrong. (T4-G001)

Some participants described acceptance strategies that buffered disappointment. They managed expectations, limited emotional exposure to uncertainty, and focused on what remained within their control, such as attending visits, following guidance, and maintaining safer practices.

#### Rebuilding Routines: Supports and Setbacks

Restarting ART required trial participants to re-establish routines and undergo the emotional adjustment of returning to taking a daily pill. Some struggled with resentment, forgetfulness, or a sense of lost progress, particularly after adapting to life off medication. Others drew on support systems, like reminders from family or shared HIV treatment adherence with partners with HIV, to ease the transition. Participants described ART restart as both practical and symbolic. Practically, they had to rebuild habits and reminder systems. Symbolically, restarting pills signaled the end of a hoped for “break,” and for some, it reactivated earlier negative memories of diagnosis, stigma, or fear of side effects.

I felt like I betrayed myself. I don’t like taking pills; I take them because I’m forced to. I feared the side effects rash, insomnia, tiredness but they didn’t happen. My body is used to them now. (T4-G041)

I felt like not taking it … on the first day I forgot to take it … it helped to tell my mom so she will remind me … I almost forgot to also set an alarm to 8 o’clock but I did. (T3-G061)

Participants often described using layered coping strategies to support adherence after restart, including disclosure to trusted family, alarms, and deliberate planning to re-anchor medication into daily schedules.

#### Looking Ahead: Hope, Purpose, and Support

Participants ultimately accepted the uncertainty of the trial and remained hopeful for future scientific breakthroughs. Ongoing support from the clinical research teams and partners, combined with a sense of contributing to something greater, eased the transition back to HIV treatment adherence.

#### LTDR Group: Prolonged Uncertainty, Growing Agency

Trial participants in the LTDR group, who remained off ART for the longest duration, described the prolonged state of uncertainty about when ART would resume. This ambiguity sometimes led to feelings of helplessness, especially as viral load monitoring shifted from weekly to biweekly. However, despite these challenges, many expressed sustained trust in the regular clinical monitoring and showed emotional readiness to remain off ART for an extended period. They adopted practical coping strategies such as setting alarms, tracking symptoms, and involving family members or partners to stay alert and prepared. Compared to other groups, their reflections suggested a deeper acceptance of the trial's uncertainty and a greater sense of agency when transitioning back to ART.

They said we would go back [on ART] when the viral load reaches a certain point … but I didn’t know when that would be, and that made me feel helpless. (T2-G001)

Now, even if I forget sometimes, I set an alarm, and my family knows I’m in the study. Going back to pills won’t be hard … it’ll just feel like returning to my old routine. (T4-G060)

For some LTDR participants, extended time off ART appeared to strengthen confidence in managing transitions. They described concrete preparation, family involvement, and a pragmatic stance toward ART restart, which signaled agency rather than resignation.

### Coping, Resilience, and Psychosocial Well-Being (T1-T4)

#### Coping and Resilience Over Time

Despite challenges, participants showed resilience and adaptive coping. Many reframed their experiences as opportunities for growth, highlighting the support received from clinical research staff and fellow trial participants. By the end of the trial, participants expressed pride in their contribution and deeper self-awareness.

At first it felt like a mountain, but now I see how much I have learned about myself. (T4-G001)

These reflections underscore the evolving nature of stress and coping during the ATI trial. While participants encountered interpersonal, procedural, and logistical challenges, many navigated them using personal strategies and reliance on the support systems available at the trial site. These trends were similar across the 3 groups categorized based on time to ART restart (ATI duration).

#### Positive Impacts of Trial Participation on HIV Literacy and Self-Management

Participants described the trial as a meaningful source of active learning rather than passive experimentation. Clear, iterative explanations from the clinical trial team enabled them to learn about viral dynamics, understand the rationale for optional procedures, and situate their own viral load outcomes within the broader goal of HIV cure research.

It's not just me being involved in the study. They’re not just using me like a guinea pig … they’re explaining very deep things that I didn’t know before … So, I feel like I’m gaining a lot of information by being in the study. (T1-G040)

Participants reflected on a range of learnings that extended beyond clinical outcomes. Many gained a deeper understanding of how everyday practices such as balanced nutrition, reducing alcohol use, and condom use can support health and viral suppression. Several described shifts in sexual behavior, including being more intentional about partner choices and practicing safer sex regardless of their partner's status. Others expressed appreciation for the opportunity to expand their knowledge about HIV and health through accessible, 2-way communication with the clinical research staff. In addition to health knowledge, some participants noted personal growth, such as increased confidence, greater self-awareness, and improved communication skills developed through trial engagement.

I learnt that balanced diet, cutting down on alcohol and using a condom contribute to keeping your viral load [being] suppressed. (T4(a)-G031)

Through the study, I learned to take care of myself, stay focused, and practice safer behavior like being with one partner and making choices that support my health. (T4-G050)

I learned about [the] lymph node incision procedure, that they can do it without causing any injury and that the scar heals like nothing has happened. (T4-G012)

Overall, participants reported that the trial enhanced their HIV-related knowledge by integrating scientific, clinical, and behavioral information, although the extent of uptake varied across participants.

#### Final Reflections and Recommendations for Future ATI Trials (T4)

Participants praised the clinical research team at FRESH for providing holistic, compassionate support throughout the trial. They emphasized how the team's personalized attention, emotional support, and clear communication built trust and kept them engaged. Many valued weekly viral load monitoring during the ATI as a key safety feature that offered reassurance and a sense of control. After viral rebound, when the protocol shifted to bi-weekly monitoring, some participants expressed a preference to continue weekly viral load checks. Their reflections suggest that maintaining flexible monitoring can better meet participants’ emotional and informational needs.

In this study, I was well taken care of. I felt love and genuine concern for my well-being, things I don’t even experience at home. The team showed humanity, compassion, and care. At home, you can be unwell, and no one might notice, but at [the trial site] they pay attention. If they see you are not okay, they talk to you, help you, and even refer you to a doctor if needed. (T4b-G011)

A couple of participants recommended procedural improvements based on their experiences. These included providing clearer dietary guidance after infusions, offering topical agents to support wound healing, and improving comfort during procedures such as blood draws and biopsies. They emphasized the value of flexible scheduling, timely symptom management, and ongoing communication with clinical research staff as essential to maintaining participation and trust.

When I first did it, they only gave me pain killers … When I went to do it again, I saw that they have improved because there were creams that they gave us. So, it helped because the wound got healed quickly this time. (T4-G070)

Participants consistently expressed enthusiasm for future participation in HIV cure-related research. Their motivations included contributing to scientific progress, improving their own health, and securing a healthier future for their children. For some, financial compensation also helped meet household needs and enhanced their sense of purpose and dignity.

For me if ever an opportunity like this arises … I would agree because … research like this, if [it] become[s] successful in the future … will help a lot now and even for future generations. (T4-G040)

It helps me stay on top of my health, and the stipend serves as a personal motivation. (T4-G012)

Lastly, a few participants recommended expanding future trials to include men. They framed this recommendation as both a scientific and practical priority. From a scientific perspective, they noted that involving people of all genders could strengthen discovery and improve the relevance of findings. From a relational perspective, they emphasized that partner understanding and shared responsibility shaped prevention practices during ATI, particularly when negotiating condom use during the ATI.

The study must mix genders … maybe there is something that they will discover. Maybe like a different gene in men, that might be helpful. (T4-G010)

I explained everything to him so during ATI we were using protection. (T4-G040)

**Figure 2.**
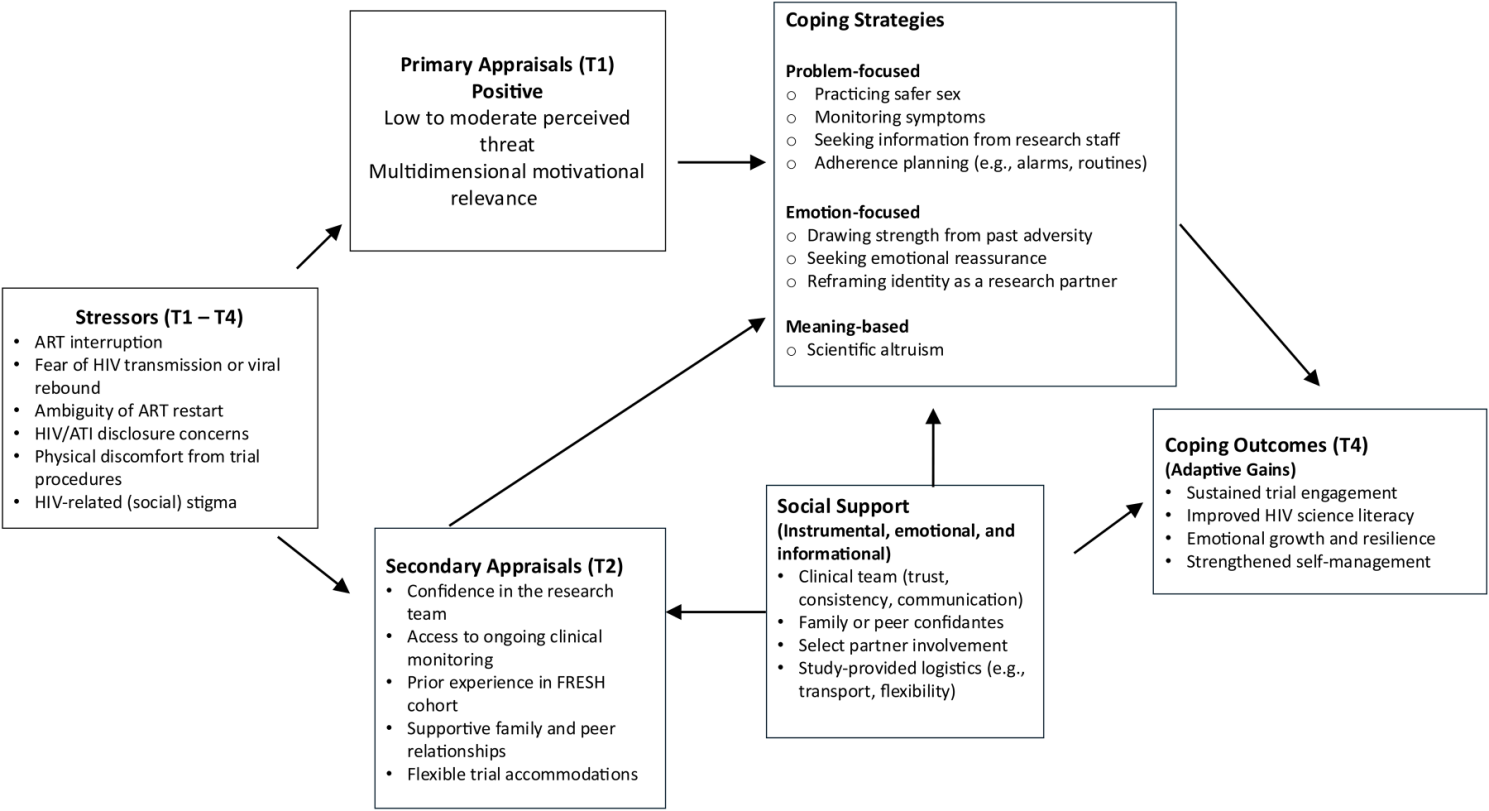
Stress-and-coping framework of participant experiences during analytic treatment interruption.

Figure 2 situates participants’ appraisals of ATI-related stressors, their mobilization of coping resources and strategies, and resulting adaptive outcomes within Lazarus and Folkman's transactional stress-and-coping framework. Supplementary Table 2 provides additional supporting quotes representing the above-mentioned themes.

## Discussion

This qualitative study is one of the first to report the lived experiences of young African WLHIV during an ATI-inclusive HIV cure trial, a population heavily affected by HIV^1^ yet underrepresented in cure research. Our findings highlight 4 key features of trial participation: participants’ initial motivations, evolving perceptions of risks and benefits, multi-level challenges encountered during the trial, and adaptive coping strategies developed over time. WLHIV joined the study to contribute to HIV cure development and reduce the burden of daily ART. Yet, they also expressed fears about stopping ART, including concerns about negative impact on health and social repercussions. These fears evolved across the ATI trial. For participants who experienced viral rebound during ATI, eventually resulting in ART restart, many expressed a growing sense of empowerment, marked by improved HIV science literacy, strengthened self-care, and renewed commitment to research. Using a stress and coping framework, we found participants navigated ATI-related uncertainty by drawing on strong trust in the frequent clinical monitoring by the trial team, developing resilience through the trial experience, finding meaning in their contribution to HIV cure research efforts. Importantly, this SBR study suggests context-specific support needs, including frequent viral load monitoring, flexible scheduling to accommodate personal responsibilities outside of the trial (caregiving and academics), disclosure-related counseling, mental health support, and physical comfort measures for trial procedures. Incorporating these participant-centered considerations may improve trial recruitment, visit and procedure adherence and mitigate potential harms associated with ATI-inclusive trials.

Participants entered the ATI trial motivated by a blend of scientific altruism and eagerness to help find an intervention capable of achieving durable ART-free viral control that would eliminate the burden of daily pill taking. These findings align with studies from high-income settings, where key motivations for trial participation included altruism, HIV stigma reduction, and relief from the clinical burdens of HIV.^4,7,17,42,43^ While scientific contribution was central, some participants noted that the study stipend eased short-term financial strain and supported their participation. In contexts of unemployment, this highlights the importance of structuring remuneration as reimbursement for participants’ time and expenses rather than as an inducement, and of assessing how payments are perceived to minimize undue influence in future HIV cure research. Alongside hope, participants also anticipated tangible risks, such as viral rebound, potential drug resistance, and the fear of transmitting HIV to partners, concerns that have been consistently documented in prior ATI research.^6,23,26,27,31,32,44^ Some feared being perceived as irresponsible due to ART nonadherence during the ATI, leading many to either disclose their trial participation to trusted individual(s) who knew their HIV status or to avoid disclosing their trial participation altogether. These strategies reflected intentional efforts to minimize social friction and emotional stress, particularly within family and partner networks.^45^ Such patterns resonate with studies in sub-Saharan Africa documenting how selective disclosure and social context shape HIV-related decision-making.^22,46^ These findings also underscore the importance of an informed consent process that addresses not only biomedical risks but also emotional and interpersonal dimensions, preparing participants to navigate potential misunderstandings and to seek psychosocial support when needed.

Participants confronted the novel stressor of interrupting ART by engaging both emotion and problem-focused coping strategies, consistent with stress and coping appraisal. Initially, women appraised ATI as both a challenge (“Can my body control the virus?”) and a threat (“What if my health deteriorates?”), but their confidence in frequent clinical monitoring by clinical research staff with whom they had a strong rapport bolstered secondary appraisals of coping resources. Trust in research staff emerged as a key stress-buffering mechanism, reducing anxiety and reinforcing adherence to the ATI trial protocol, as seen in similar ATI-inclusive studies.^23,25,47,48^ Emotion-intensive coping took the form of positive reappraisal: participants found meaning in contributing to HIV cure science, which strengthened resilience and counteracted feelings of vulnerability. Simultaneously, they employed problem-focused coping that included condom use, partner HIV testing, and vigilant clinic attendance to manage transmission risk and health uncertainties, aligning with recommendations from youth-centric HIV research.^49–51^ Many also navigated disclosure with thoughtfulness, confiding only in those they perceived as emotionally safe. These interpersonal coping strategies helped protect their autonomy while managing potential community misinterpretation of stopping ART. Together, these findings underscore the value of including integrated psychosocial and biomedical supports such as peer networks and counseling in ATI-inclusive trial protocols. Although we anticipated differences in coping by ATI duration, participants across subgroups employed broadly similar strategies. For those in the LTDR group, reflections suggest that resilience and emotional readiness were shaped more by consistent support, such as frequent monitoring and trusted staff relationships, than by the duration of time off ART. Similar observations were reported in a San Francisco ATI interventional study, where participants described a sense of being “in limbo” during the period of prolonged off-ART viral suppression, underscoring that structured, person-centered support can buffer uncertainty and sustain engagement even during extended ATIs.^17^

Partner dynamics also played a critical role, as the ATI required navigating issues of sexual safety and trust. Some participants described supportive relationships, including participation of partners in informational sessions or collaborative agreement on prevention strategies (eg, PrEP or condom use), which appeared to strengthen emotional resilience.^52^ These anticipated complexities were also noted among participants in the acute arm of the FRESH cohort who were eligible but did not enroll in the ATI trial, where women described how partner reactions and the need for disclosure would shape their decision-making around trial participation.^10^ These findings also align with the Social Support Theory, which emphasizes the importance of emotional, informational, and practical support in navigating chronic and stigmatized health conditions.^53^

Moreover, participants described logistical challenges related to ATI trial participation. The intensive protocol, frequent clinic visits, venipuncture, and periodic prolonged procedures created fatigue and disruptions to daily responsibilities. WLHIV also faced difficulty coordinating trial visits with academic and caregiving obligations, despite the trial's reimbursements and flexible accommodations. These burdens were especially pronounced for younger women with fewer resources, consistent with prior evidence on barriers to trial retention in resource-limited settings.^54–56^ Some women experienced pain or discomfort from repeated blood draws. Difficult venous access also emerged as a concern, with participants attempting hydration, exercise, or weightlifting to prepare. These challenges may reflect broader sex-based physiological differences, as women's smaller veins can complicate apheresis procedures and simple phlebotomy.^57^ Addressing such procedural burdens through improved preparation and support could enhance participant safety and retention. Despite these hurdles, no participants withdrew from the ATI trial; a testament to their resilience and to the trusting relationships formed with the clinical research team.

Our findings align with emerging best practices that call for women-centered, holistic clinical trial designs that go beyond generic psychosocial care to explicitly address women's personal responsibilities outside of the trial, layered stigma, bodily autonomy, and unequal power dynamics in relationships.^58^ Based on participant narratives, we developed a set of actionable, phase-specific recommendations to inform future ATI trial design (Table 2). These include enhancements to informed consent, ongoing mental health and psychosocial support, partner engagement strategies, procedural comfort measures, and broader logistical accommodations. Such adaptations are essential to designing trials that are optimally ethical, inclusive, and supportive of participation and retention, especially among young WLHIV in resource-constrained settings.

**Table 2.** Actionable Recommendations to Improve ATI Trial Design That Emerged from our Findings.

Trial Phases	Recommendations	Implementation Examples
Informed consent and screening	Strengthen informed consent communication	Use simplified, phase-specific materials (eg, visual aids, scripts) that clearly explain ATI-related steps, risks, and what to expect.
	Support disclosure decision-making	Offer one-on-one coaching and optional joint counseling to help participants choose a trusted confidante (partner, family member or any social connection) and practice disclosure.
Pre-ATI	Embed early psychosocial check-ins	Schedule counseling or peer support at enrollment and pre-ATI phase to assess readiness and address anxiety, HIV-related stigma, relational and other stressors.
	Provide partner engagement options	Offer informational materials or flexible opportunities for partner involvement, such as optional visits, not mandatory disclosures.
ATI phase	Ensure frequent viral load monitoring and feedback	Maintain weekly viral load testing (can be variable depending on the nature of the trial, ATI phase and/or participant preferences) and real-time communication to support participants’ sense of safety and control.
	Improve procedural comfort	Rotate venipuncture sites, help participants pre-hydrate, apply topical anesthetics for biopsies, and provide wound care and follow-up.
	Address scheduling and transport barriers	Offer transport vouchers, childcare, and flexible visit hours (early/late/weekend); send reminders via text or calls.
Post-ATI and ART restart	Provide re-initiation counseling and adherence support	Discuss emotional responses to restarting ART, offer adherence tools (eg, alarms, family reminders), and normalize adjustment period.
Ongoing support across ATI trial phases	Foster peer support throughout	Organize in-person or virtual peer groups frequently; emphasize confidentiality and shared learning.
	Enhance health literacy resources	Distribute translated ATI guides (in local language—eg, *is*iZulu) and short videos covering trial procedures, viral rebound, self-care tips, and emergency contacts, ensuring that all materials avoid stigmatizing language (eg, references to HIV).
	Maintain longitudinal engagement post-trial	Conduct follow-up calls or texts for at least 3 months and/or 1-year post-trial to monitor well-being and reinforce linkages to HIV treatment and care.

Abbreviations: ATI, analytical treatment interruption; ART, antiretroviral therapy.

## Limitations

We interpret these findings considering several limitations. First, this was a single-site study with a modest sample of young women in one setting. As with most qualitative research, our aim was depth and contextual understanding rather than statistical generalization. Readers should consider how the FRESH cohort context, local social norms, and clinical trial procedures may shape the transferability of findings to other settings and populations. Second, participants had long-term involvement in the FRESH program, which may have influenced their willingness to participate and what they were comfortable sharing. We sought to mitigate this by using trained socio-behavioral research staff, conducting interviews in private spaces, and emphasizing confidentiality and voluntary participation. Third, we relied on self-reported accounts, which may be affected by recall and social desirability, particularly for sensitive topics such as disclosure and partner protections. Fourth, the focus on women limits insights into the experiences of men and nonbinary individuals. Fifth, we did not include a comparison group of women who did not undergo ATI, so we cannot disentangle which experiences were specific to ATI versus broader trial participation. Finally, while interview guides supported consistency across timepoints, they may have constrained unanticipated lines of inquiry, although interviewers used open-ended probes and the team reviewed emerging insights throughout analysis. Future multi-site, mixed-methods studies with more diverse cohorts and open-ended inquiry are needed to validate and build upon these findings.

## Conclusions

Our findings underscore that ATI trials that enroll young WLHIV in HIV high-burden resource-limited settings are strengthened by integrated, participant-centered models of care that foreground psychosocial support, partner engagement, and operational flexibility. Inclusion of routine mental health monitoring, tailored risk-reduction counselling, and logistical accommodations within ATI-inclusive trial protocols can contribute to successful HIV cure trial implementation. As the field advances toward scalable cure strategies, these data advocate for human-centered trial frameworks that reconcile scientific objectives with the lived realities of those most affected by HIV and in need of effective HIV cure or durable ART-free control options.

## Supplemental Material

sj-docx-1-jia-10.1177_23259582261423985 - Supplemental material for Stress and Coping During an HIV Cure-Related Trial with an Analytical Treatment Interruption: A Qualitative Assessment of the Experiences of Young Women in Durban, South Africa Supplemental material, sj-docx-1-jia-10.1177_23259582261423985 for Stress and Coping During an HIV Cure-Related Trial with an Analytical Treatment Interruption: A Qualitative Assessment of the Experiences of Young Women in Durban, South Africa by Ali Ahmed, Miranda Hill, Krista L. Dong, Mzwakhe Wiseman Ngcobo, Ayanda Zulu, Ntombifuthi Langa, Luyanda Maphalala, Vanessa Pillay, Maud Mthembu, Whitney Tran, Rachel Lau, Jamila K. Stockman, Thumbi Ndung’u and Karine Dubé in Journal of the International Association of Providers of AIDS Care (JIAPAC)

sj-docx-2-jia-10.1177_23259582261423985 - Supplemental material for Stress and Coping During an HIV Cure-Related Trial with an Analytical Treatment Interruption: A Qualitative Assessment of the Experiences of Young Women in Durban, South Africa Supplemental material, sj-docx-2-jia-10.1177_23259582261423985 for Stress and Coping During an HIV Cure-Related Trial with an Analytical Treatment Interruption: A Qualitative Assessment of the Experiences of Young Women in Durban, South Africa by Ali Ahmed, Miranda Hill, Krista L. Dong, Mzwakhe Wiseman Ngcobo, Ayanda Zulu, Ntombifuthi Langa, Luyanda Maphalala, Vanessa Pillay, Maud Mthembu, Whitney Tran, Rachel Lau, Jamila K. Stockman, Thumbi Ndung’u and Karine Dubé in Journal of the International Association of Providers of AIDS Care (JIAPAC)

sj-docx-3-jia-10.1177_23259582261423985 - Supplemental material for Stress and Coping During an HIV Cure-Related Trial with an Analytical Treatment Interruption: A Qualitative Assessment of the Experiences of Young Women in Durban, South Africa Supplemental material, sj-docx-3-jia-10.1177_23259582261423985 for Stress and Coping During an HIV Cure-Related Trial with an Analytical Treatment Interruption: A Qualitative Assessment of the Experiences of Young Women in Durban, South Africa by Ali Ahmed, Miranda Hill, Krista L. Dong, Mzwakhe Wiseman Ngcobo, Ayanda Zulu, Ntombifuthi Langa, Luyanda Maphalala, Vanessa Pillay, Maud Mthembu, Whitney Tran, Rachel Lau, Jamila K. Stockman, Thumbi Ndung’u and Karine Dubé in Journal of the International Association of Providers of AIDS Care (JIAPAC)
