# Supplementary material for: Stress and Coping During an HIV Cure-Related Trial with an Analytical Treatment Interruption: A Qualitative Assessment of the Experiences of Young Women in Durban, South Africa
Source: J Int Assoc Provid AIDS Care. 2026 Feb 13;25:23259582261423985. doi: 10.1177/23259582261423985 (PMC12905108; doi:10.1177/23259582261423985)
Supplement: sj-docx-1-jia-10.1177_23259582261423985 - Supplemental material for Stress and Coping During an HIV Cure-Related Trial with an Analytical Treatment Interruption: A Qualitative Assessment of the Experiences of Young Women in Durban, South Africa [file sj-docx-1-jia-10.1177_23259582261423985.docx]

**Supplementary File 1: Consolidated Criteria for Reporting Qualitative Studies (COREQ): 32-item checklist**

**Manuscript Title:** Stress and Coping during an HIV Cure-Related Trial with an Analytical Treatment Interruption: A Qualitative Assessment of the Experiences of Young Women in Durban, South Africa

| **Item** | **Guide question or description** | **Reported in manuscript (section) or action** |
| --- | --- | --- |
| **Domain 1: Research team and reflexivity** |  |  |
| Personal characteristics |  |  |
| 1. Interviewer/facilitator | Which author conducted the interview or focus group? | Methods, Data collection: Interviews were conducted by trained sociobehavioral research (SBR) staff (M W N, A Z, N L) |
| 2. Credentials | What were the researcher's credentials? (e.g., PhD, MD) | Methods, Research team: Interviewers and qualitative analysts held relevant academic and clinical qualifications (e.g., bachelor’s, MPhil, PhD, MD). |
| 3. Occupation | What was their occupation at the time of the study? | Methods, Data collection: At the time of the study, interviewers were research staff affiliated with the study team. |
| 4. Gender | Was the researcher male or female? | Methods, Research team: The research team included interviewers of different genders. |
| 5. Experience and training | What experience or training did the researcher have? | Methods: Methods, Data collection / Data analysis: Interviewers were trained in qualitative interviewing and study procedures and worked under supervision with regular debriefing and consensus processes. |
| Relationship with participants |  |  |
| 6. Relationship established | Was a relationship established prior to study commencement? | Methods: Participants and Recruitment (FRESH context); Limitations (prior involvement may influence disclosure) |
| 7. Participant knowledge of the interviewer | What did participants know about the interviewer? | Not explicitly reported; implied via consent process and SBR team introduction |
| 8. Interviewer characteristics | What characteristics were reported about the interviewer/facilitator? | Methods: Interviewer education, expertise and purpose of study reported. |
| **Domain 2: Study design** |  |  |
| Theoretical framework |  |  |
| 9. Methodological orientation and theory | What methodological orientation underpinned the study? | Methods: Data Analysis (framework analysis; transactional model of stress and coping) |
| Participant selection |  |  |
| 10. Sampling | How were participants selected? | Methods: Participants and Recruitment (women enrolled in ATI trial recruited to SBR) |
| 11. Method of approach | How were participants approached? | Methods: Participants and Recruitment (introduced at screening; referred to independent SBR team) |
| 12. Sample size | How many participants participated? | Methods: Study Design and Setting; Results: Participant characteristics |
| 13. Non participation | How many people refused or dropped out and why? | Results/Participant flow: one declined qualitative study (noted in manuscript); reasons not collected |
| Setting |  |  |
| 14. Setting of data collection | Where was the data collected? | Methods: Data Collection (private room at FRESH site) |
| 15. Presence of non participants | Was anyone else present besides the participants and researchers? | Not reported; assumed private interviews; will add explicit statement |
| 16. Description of sample | What are the important characteristics of the sample? | Results/Table 1 (demographics and clinical characteristics) |
| Data collection |  |  |
| 17. Interview guide | Were questions, prompts, guides provided? | Methods: Interview Guide (semi structured guide; timepoint specific) |
| 18. Repeat interviews | Were repeat interviews carried out? How many? | Methods: Study Design and Setting (four IDIs at T1 to T4) |
| 19. Audio/visual recording | Did the research use audio/visual recording? | Methods: Data Collection (audio recorded) |
| 20. Field notes | Were field notes made during or after interviews? | Yes |
| 21. Duration | What was the duration of the interviews? | Methods: Data Collection (30 to 90 minutes) |
| 22. Data saturation | Was data saturation discussed? | Methods: Data Analysis (thematic sufficiency statement added) |
| 23. Transcripts returned | Were transcripts returned to participants for comment or correction? | Not reported; participant validation checks not conducted (added in Methods) |
| **Domain 3: Analysis and findings** |  |  |
| Data analysis |  |  |
| 24. Number of data coders | How many data coders coded the data? | Methods: Data Analysis (three coders) |
| 25. Description of the coding tree | Did authors provide a description of the coding tree? | Partially (a priori codebook described) |
| 26. Derivation of themes | Were themes identified in advance or derived from the data? | Methods: Data Analysis (a priori plus inductive refinement) |
| 27. Software | What software, if applicable, was used to manage data? | Not specified; framework matrices described. |
| 28. Participant checking | Did participants provide feedback on the findings? | Methods: Data Analysis (member checks not conducted; alternative validation strategies described such as double-checking transcripts and team-based review) |
| Reporting |  |  |
| 29. Quotations presented | Were participant quotations presented to illustrate themes? | Results (quotes throughout; participant IDs and timepoints) |
| 30. Data and findings consistent | Was there consistency between the data presented and the findings? | Results and Discussion (themes supported by quotes) |
| 31. Clarity of major themes | Were major themes clearly presented? | Results (themes and subthemes clearly labeled) |
| 32. Clarity of minor themes | Is there a description of diverse cases or minor themes? | Results (contrasting appraisals and subgroup comparisons; LTDR, DR, ER) |
