# Supplementary material for: Stress and Coping During an HIV Cure-Related Trial with an Analytical Treatment Interruption: A Qualitative Assessment of the Experiences of Young Women in Durban, South Africa
Source: J Int Assoc Provid AIDS Care. 2026 Feb 13;25:23259582261423985. doi: 10.1177/23259582261423985 (PMC12905108; doi:10.1177/23259582261423985)
Supplement: sj-docx-2-jia-10.1177_23259582261423985 - Supplemental material for Stress and Coping During an HIV Cure-Related Trial with an Analytical Treatment Interruption: A Qualitative Assessment of the Experiences of Young Women in Durban, South Africa [file sj-docx-2-jia-10.1177_23259582261423985.docx]

**Supplementary Table 2: Additional Quotes – Trial Participants Experiences on HIV Cure Research with ATIs (Durban, South Africa, 2022 – 2025)**

| **Themes** | **Participants** | **Quotes** |
| --- | --- | --- |
| **Motivations and Decision Making at Screening (T1)** | | |
| Scientific altruism | T1-G010 | *I am a young woman living with HIV… maybe I have antibodies that… could help get an antidote or something, to stop the virus.* |
|  | T1-G031 | *I joined to help other people, so that they can find HIV cure.* |
| Desire for reduced ART burden | T1-G050 | *I wanted to see if this thing of bnAbs [broadly neutralizing antibodies] will work for me to be able to live without having to take ARV’s [antiretrovirals].* |
|  | T1-G070 | *I joined because I wish that one day, I could stop taking pills everyday maybe find something that will last longer.* |
|  | T1-G011 | *Maybe [bnAbs] will suppress in a way that I live without pills.* |
| Interest in HIV knowledge | T1-G071 | *I wanted to know more about HIV and see if there is a possibility to find cure. Also to find out how can I disclose my status to partner.* |
|  | T1-G051 | *I do not have people that I can talk to about my situation… I believed if I joined, I would get strength to tell my family.* |
| Financial compensation | T1-G041 | *The reason I joined… I saw it will be helpful to me, even about the stipend I heard about it, after I have already joined.* |
|  | T1-G090 | *I benefit a lot from getting money because I am not working, and I do nothing for a living, so I get to buy myself cosmetics and other stuff.* |
| Informed decision making | T1-G040 | *They’ve told me everything… the only risk… is the one that involves the ATI period where the virus could spiral out of control.* |
| Trust in study setting | T1-G030 | *Listening to the FRESH staff explaining to me about this pill made it easy for me to agree to join the study.* |
|  | T1-G010 | *When I found out that I was HIV positive [had HIV] … I had the support of FRESH, so it was really easy to participate in [trial].* |
| **Evolving Appraisals of Stressors, Risks, and Benefits (T1 – T4)** | | |
| Viral rebound illness | T1-G020 | *Worried that… I will get sick in the absence of a pill in my body.* |
|  | T2-G041 | *I’m not on ATI yet, but I already see it as a problem, having to stop my pills for the research feels wrong. I’m scared my viral load will go up, like all our effort was for nothing. I told my partner I needed space, but I didn’t tell him the real reason. I just want to deal with this alone, focus on me, and not worry about anyone else right now.* |
| Fear of investigational drug safety | T2-G060 | *They told me that there is nothing harmful, so that comforted me because at first, I had “what ifs” on my mind, thinking that normally these pills are taken every day. Now if I am stopping “what if” so I was worried about the side effects of these study drugs and if my body will respond well or not.* |
| Partner HIV transmission risk and risk negotiation | T1-G070 | *You can easily infect [pass HIV to] your partner or maybe he can also re-infect [pass HIV to] you.* |
|  | T1-G060 | *A person that you are dating you must use protection all the time, so I think it’s easy for a person who is already using condoms when having sexual intercourse. But for me, since I’ve joined the study, it’s going to be a bit difficult because most of the time my partner and I do not use a condom. So obviously if suddenly, I tell him to use a condom it will be difficult. I need to come up with a solid reason why… I will have to stop taking pills, there is a possibility of me infecting [passing HIV to] him.* |
|  | T3-G020 | *I once heard that once you stop ARV’s [antiretrovirals] or not adherent they end up not helping. Yes, but this was just me thinking I never shared this with anyone here at FRESH to get better explanation.* |
|  | T3-G071 | *If it happens that you get pregnant while on the study, that is risky to the baby.* |
| Family support | T1-G030 | *My mother was scared when I told her I’d stop ARVs. She thought I’d get sick again. But after I explained how they’d monitor me and that it’s for research, she said she’d pray for me and support me.* |
|  | T1-G070 | *My cousin said, “It’s good you’re doing this”. People like us need to be part of finding a cure.’ That gave me strength.* |
|  | T2-G050 | *My aunt was confused why I’d stop treatment. I told her to speak with the counselor. After that, she was okay with it.* |
| Emotional risk and disclosure | T1-G031 | *It is very hard to tell people who they did not have an idea that you are sick.* |
|  | T2-G061 | *After doing lymph nodes, you do not feel good so people might ask what is happening, now I will have to explain myself.* |
|  | T1-G010 | *I’m just gonna talk about emotional [emotions]. Because sometimes you invest your emotions too much… and then it doesn’t work… you must tell yourself that it’s 50-50… so that you can protect your feelings.* |
|  | T2-G051 | *There can be side effects that are not normal… we respond in different ways.* |
| Feeling secure | T4-G040 | *It helped me in a lot of things… the stipend… boosted my confidence.* |
|  | T4b-G060 | *So far so good, my bloods are okay and everything.* |
|  | T4b-G050 | *So far, I am okay… my virus is still suppressed… that’s what I liked the most.* |
| Trust and health support | T3-G001 | *They care for us… those are the things that are not done in the government hospitals.* |
|  | T4-G020 | *Even if I was sick with my personal things, FRESH will go out of the way and pay for treatment… it helped me in many things.* |
|  | T3b-G031 | *I only got sick once with an abscess… Other than that, I haven’t experienced any serious illnesses.* |
|  | T3-G030 | *The nurses explained each step and reminded me that I could stop anytime… it helped me feel safer.* |
| **Perceptions of ATI and ART Restart (T3 – T4)** | | |
| Anxiety due to trial outcome uncertainty | T2-G001 | *They explained that when my viral load goes up, they’ll let me know. But I didn’t know how high it had to be, or how long it would take.* |
|  | T2-G090 | *I didn’t know how long I’d be off ART… not knowing made it worse.* |
|  | T2-G060 | *I just kept wondering, what if I get sick before they tell me to go back [on ART]?* |
| Informed vigilance | T2-G051 | *I kept checking my body, how I felt, if I was getting sick… I was more alert than ever.* |
|  | T3-G030 | *I started noting everything headaches, sleep, appetite so I could tell the nurses.* |
| Mixed emotions at ART restart | T2-G020 | *When they said I should start again, I was relieved. I felt like I had my weapon back.* |
|  | T2-G060 | *It was hard… I had hoped maybe I would last longer off pills. When I had to go back, I felt like I failed.* |
|  | T4b-G011 | *I’m still on ATI, I’m happy, but when I get back on the pills, I won’t have a problem. We were told that the study is not guaranteed, the treatment was not going to get rid of the virus in our bodies.* |
|  | T4-G010 | *I was not happy [about viral load going up], after all I was already used to not taking it, I had freedom. I had hope that we are going to be cured, and then suddenly, they tell me my viral load is up I must take my treatment again.* |
|  | T4-G031 | *I felt bad that I did not complete the entire study process, but I was not deeply hurt about restarting my treatment because I knew I still had the virus in my blood. I understood that this was just a trial, they were still testing it and were not sure if it would be successful.* |
| Acceptance and altruistic framing | T2-G040 | *Even if it didn’t work for me, I was part of something bigger… maybe next time it will be different.* |
| Informational support | T3-G010 | *If I say that I hurt me I would be lying because like as we are going for counselling here at FRESH they always make sure to remind us that this is not 100% sure we are trying and you know it’s a trial so it can come back, it cannot come back maybe you’re a post controller and stuff like that, yes because after that six months it came up and then it went down and then few weeks it went up again so yeah.* |
| Emotional impact of ART restart | T4-G0141 | *I felt like I betrayed myself… I felt like if I gave myself a chance to finish, it would have been better.* |
| Physical adaptation to ART restart | T3-G061 | *The first day I forgot to take it… I was no longer used to taking it.* |
| Partner and family reminders | T4-G090 | *My partner also reminds me, “have you taken your pills” even when I have forgotten, when he reminds me, I will be like “oh I almost forgot”. Then I will go and get it.* |
| **Coping, Resilience, And Psychosocial Well-Being (T1 – T4)** | | |
| Interpersonal and disclosure-related challenges | T1-G090 | *I don’t know what his problem is but ever since I joined here, I told him that it’s either he wears a condom, or we don’t have sex because I am protecting… he doesn’t want to use a condom.* |
|  | T1-G071 | *Challenges from home are the one’s difficult to handle… I feel in whatever I do, I must do it privately… Another challenge is with my boyfriend, when it comes to taking my medication, it is a difficult situation.* |
| Challenges related to trial procedures | T3b-G011 | *While I was on the screen process, when they had to take my blood, my veins would not allow them… They said I must exercise, drink water, lift weight, that also helps.* |
|  | T4-G020 | *Obviously taking blood is not something you can get used too, and the thing that I had problem with was when I was put in that machine, I was near to quitting the study because I had a very terrible headache after.* |
| Logistical and scheduling challenges | T4b-G091 | *I moved to another location and yes, traveling is expensive, and I’m not willing to pay out of my pocket just to get to the clinic.* |
|  | T3-G012 | *Uhm, stipend is very benefiting me as I am not working, it boosts me in a way that I can buy food to eat.* |
|  | T2-G051 | *Maybe if it happens that I get a job, that will be a challenge for the study, that means I will have to quit. Other than that, I do not see any other challenges because with studying, I am studying online, everything I do it online so I do not think there can be challenges.* |
| Coping and resilience over time | T3-G030 | *Even when it was tough, I knew I could talk to the nurses. They helped me stay calm.* |
|  | T4-G010 | *They took me from home to the hospital… performed surgery… FRESH paid for my transport… supported me through the whole ordeal.* |
|  | T1-G051 | *I do not think there can be challenges, it is just that we do not know, anything can happen as time goes on. I really do not believe there can be challenges, because with everything you do, you consult with your ancestors.* |
| **Positive Impacts of Trial Participation on HIV Literacy and Self-Management** | | |
| Reframing ART adherence and resilience | T4-G010 | *I would say there are clever people out there, for the fact that they developed something like bnAbs. Which can maintain our viral load for such a long time, so with knowledge I learnt that there is nothing you cannot do, so I think there is progress little by little.* |
| Empowerment through learning | T4-G050 | *I don’t think the way they explain HIV here you can find it in the hospital, here they make you understand that you can live with the virus and have it suppressed if you are taking your ARV’s correctly, they explain everything and make you realize that you can live with the virus.* |
|  | T1-G041 | *Oh, there is a lot I have learned because before I did not know where you can find HIV in a body. They teach us about all those things, it is not like training, but they explain things to us, and you get to ask questions. They also ask us questions and ask us if this or that.* |
|  | T4-G060 | *All I can say is that I saw the importance of using a protection when we are having sex because mainly you must consider your partner in so many things even if he is also positive or not, or even if he is taking pills or not protection prevents you from many things in every time.* |
|  | T4-G051 | *I’ve learned the most is the importance of communication. As a naturally quiet person who tends to avoid speaking up, being part of this study has helped me become more confident in expressing myself and engaging with others. I’ve learned to open up and share my thoughts, breaking the habit of staying quiet for too long.* |
| Excited to challenge stigma | T1-G071 | *People I am living with are judgmental to people who are in this situation. They will talk bad about them so I will be able to change their mindset, and the way they talk.* |
| Peer support | T4-G051 | *When we’re all together, we can discuss our shared experiences and support one another. Plus, financial compensation brings us joy.* |
|  | T4-G060 | *I saw the importance of using protection when we are having sex… protection prevents you from many things.* |
| **Final Reflections and Recommendations for Future ATI Trials (T4)** | | |
| Nutritional support | T4-G080 | *They should guide us on what to eat, especially after taking bnAbs.* |
| Wishing for more opportunity | T4-G012 | *I wish they had increased visiting days to at least twice a week. Unfortunately, I will not be around to benefit from it, but if I were staying two days it would have been ideal. That way, I’d feel truly productive, since no one can simply hand me that amount of money, it is not easily found.* |
|  | T4b-G011 | *I’d say that this idea of coming every other week should change. We must come every day so they can monitor us regularly. Also, if we are given this treatment, they should provide a higher dose. If we stay in one place, they might see how it works, because we might be living our lives recklessly, and that could affect our medication* |
| Holistic support | T4b-G091 | *The nursing staff really took good care of us; they supported us emotionally, physically, and in every way. Even if I was stressed… I could talk to them [FRESH nurses], and they would give me good advice.* |
|  | T4-G020 | *I still want to continue because of the care and good treatment I received from the FRESH staff.* |
|  | T4-G040 | *Well, as I have said on science side, I did get a lot of information and on how do they work.* |
| Future orientation | T4-G091 | *What we are doing is no longer just about us but for those who will come after us, in the hope that a cure will be found.* |
|  | T4-G040 | *It makes me feel proud to know that at some point I am going to be named as one of the people involved in such a big medical breakthrough.* |

T=Time point; At T3 and T4 for some participants more than one interview was done and referred as T3a or T3b and likewise for T4.
