# Supplementary material for: Stress and Coping During an HIV Cure-Related Trial with an Analytical Treatment Interruption: A Qualitative Assessment of the Experiences of Young Women in Durban, South Africa
Source: J Int Assoc Provid AIDS Care. 2026 Feb 13;25:23259582261423985. doi: 10.1177/23259582261423985 (PMC12905108; doi:10.1177/23259582261423985)
Supplement: sj-docx-3-jia-10.1177_23259582261423985 - Supplemental material for Stress and Coping During an HIV Cure-Related Trial with an Analytical Treatment Interruption: A Qualitative Assessment of the Experiences of Young Women in Durban, South Africa [file sj-docx-3-jia-10.1177_23259582261423985.docx]

**Supplementary File: Experiences of Young Women Undergoing Analytical Treatment Interruption in a HIV Cure-Related Trial in Durban, South Africa**

Thank you for participating in this interview. This study will help us understand how we can better engage women enrolled in HIV cure-related research.

This interview guide was used to explore participants’ experiences at four longitudinal timepoints (T1–T4) during the ATI trial. T1 refers to the baseline (pre-ATI) interview; T2 was conducted immediately prior to ATI initiation; T3 during or shortly after ATI; and T4 at the end of study or early discontinuation.

**Motivations, Decision-Making, and Expectations**

T1-Q1. Why did you decide to participate in the clinical trial?

T1-Q2. What parts of the clinical trial were most important to you and why?

T1-Q4. How easy was it for you to arrive at the decision to participate in the trial?

**Perceived Risks and Benefits**

T1-Q5. In your view, what are the risks a person must consider when participating in a trial like this?

T1-Q6. What are the benefits and challenges you see from participating in this trial?

T2-Q1/T3-Q1/T4-Q1. Do you feel you are benefiting from participating in the trial?

**Perceptions of ATI and ART Restart**

T1-Q7. What are your thoughts and feelings about pausing ARVs during the trial (ATI)?

T2-Q2. How do you feel about the upcoming ATI? What worries you the most?

T3-Q2. Describe your feelings about stopping your ARVs during the ATI.

T3 – Additional Prompts:

What sorts of feelings did you experience during the ATI?

How did you deal with those feelings?

Did you discuss them with anyone (e.g., family, friends)? What were those conversations like?

Did you experience any changes in your physical or emotional health?

How did it feel to restart ARVs (or how do you anticipate it will feel)?

T4-Q2. How did you feel about pausing your ARVs during the ATI?

T4-Q3. How did you feel about restarting your ARVs?

T4-Q4. Would you consider participating in an ATI again?

**Anticipated and Encountered Challenges**

T1-Q8. Describe any challenges you have faced in this or previous studies. How did you manage them?

T1-Q9. What challenges do you think you might face in this trial and why?

T2-Q3. What challenges have you experienced so far? How have you managed them?

T2-Q4. What challenges do you think might arise later in the trial?

T3-Q4 / T4-Q5. What challenges did you face during the trial, and how did you deal with them?

**Knowledge Gained and End-of-Study Reflections**

T1-Q10. How do you feel about the knowledge you will gain from participating in this trial?

T4-Q6. What did you learn from your participation in the trial?

T4-Q7. Would you consider participating in another study like this one?

T4-Q8. What recommendations do you have to improve future studies?

T4-Q9. Is there anything else you would like to share about your participation?

Thank you for taking the time to answer these questions. Your participation in this interview greatly contributes to advancing our knowledge of how we can best engage women in HIV cure-related research.
